# Intein-based thermoregulated meganucleases for containment of genetic material

**DOI:** 10.1093/nar/gkad1247

**Published:** 2024-01-05

**Authors:** Gary W Foo, Christopher D Leichthammer, Ibrahim M Saita, Nicholas D Lukas, Izabela Z Batko, David E Heinrichs, David R Edgell

**Affiliations:** Department of Biochemistry, Schulich School of Medicine and Dentistry, London, Ontario N6A 5C1, Canada; Department of Biochemistry, Schulich School of Medicine and Dentistry, London, Ontario N6A 5C1, Canada; Department of Biochemistry, Schulich School of Medicine and Dentistry, London, Ontario N6A 5C1, Canada; Department of Biochemistry, Schulich School of Medicine and Dentistry, London, Ontario N6A 5C1, Canada; Department of Microbiology and Immunology, Schulich School of Medicine and Dentistry, London, Ontario N6A 5C1, Canada; Department of Microbiology and Immunology, Schulich School of Medicine and Dentistry, London, Ontario N6A 5C1, Canada; Department of Biochemistry, Schulich School of Medicine and Dentistry, London, Ontario N6A 5C1, Canada

## Abstract

Limiting the spread of synthetic genetic information outside of the intended use is essential for applications where biocontainment is critical. In particular, biocontainment of engineered probiotics and plasmids that are excreted from the mammalian gastrointestinal tract is needed to prevent escape and acquisition of genetic material that could confer a selective advantage to microbial communities. Here, we built a simple and lightweight biocontainment system that post-translationally activates a site-specific DNA endonuclease to degrade DNA at 18°C and not at higher temperatures. We constructed an orthogonal set of temperature-sensitive meganucleases (TSMs) by inserting the yeast VMA1 L212P temperature-sensitive intein into the coding regions of LAGLIDADG homing endonucleases. We showed that the TSMs eliminated plasmids carrying the cognate TSM target site from laboratory strains of *Escherichia coli* at the permissive 18°C but not at higher restrictive temperatures. Plasmid elimination is dependent on both TSM endonuclease activity and intein splicing. TSMs eliminated plasmids from *E. coli* Nissle 1917 after passage through the mouse gut when fecal resuspensions were incubated at 18°C but not at 37°C. Collectively, our data demonstrates the potential of thermoregulated meganucleases as a means of restricting engineered plasmids and probiotics to the mammalian gut.

## Graphical Abstract

**Graphical Abstract:**
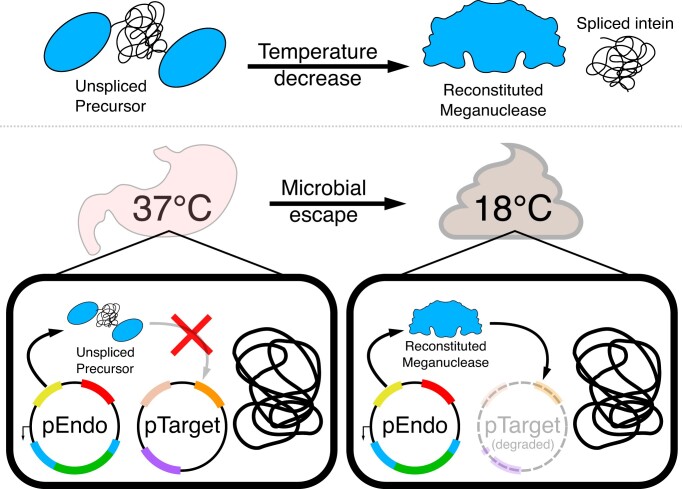

## Introduction

Biocontainment strategies to limit the spread of genetically modified microorganisms and associated genetic material are an essential component of synthetic biology research (1,2). In recent years, engineered bacterial probiotic strains have been designed to act as next-generation therapeutics to restore microbial homeostasis in dysbiotic conditions in the human microbiome (3–5). For engineered strains that carry plasmids or other accessory elements, robust biocontainment systems are needed to prevent the escape and dissemination of recombinant genetic material before widespread adoption of engineered strains in human medicine, or other applications where biocontainment is critical. In particular, DNA released to the environment could be acquired by naturally competent bacteria or spread by mechanisms that promote lateral gene transfer, potentially conferring a selective advantage depending on the integrity and information carried on synthetic molecules (6–8). Biocontainment systems must therefore be able to sufficiently degrade recombinant genetic material and limit acquisition by microbial communities (9–12).

Current biocontainment strategies that rely on auxotrophic dependencies or self-killing systems that are regulated by multi-layered genetic circuits and external signalling molecules are generally designed for laboratory conditions (9,13–16). For applications in the mammalian gastrointestinal (GI) tract, some of the required signalling molecules are either potential carbon sources or restricted by bioavailability and thus not optimized to limit environmental escape of genetic material after cell death. Biocontainment is particularly relevant for genetically modified strains that deliver engineered plasmids or conjugative systems (17–21) to the mammalian GI tract to prevent uptake of foreign sequences that could provide a selective advantage, such as antibiotic resistance genes. In contrast, kill switches comprised of site-specific or non-specific DNA endonucleases that promote DNA degradation through the RecBCD pathway (22) are well suited to limit the spread of genetic material (10–12). Previously, type II restriction enzymes such as EcoRI (23) were used in endonuclease-based kill-switches, and a temperature-sensitive dCas9 variant active at 29°C but not 37°C was also developed (24). Using Cas9 as a biocontainment strategy in microbiomes precludes its use in editing or regulating gene expression because there is no mechanism for Cas9 to distinguish between multiplexed guideRNAs. This issue could be overcome by using orthogonal Cas9 variants with different protospacer associated motif (PAM) and crRNA scaffold requirements (25), but this increases the complexity of the system, the potential for escape mutants, and the potential for off-target guideRNA effects as single mutations can change target specificity, or inactivate the guideRNA (26–29).

**Figure 1.**
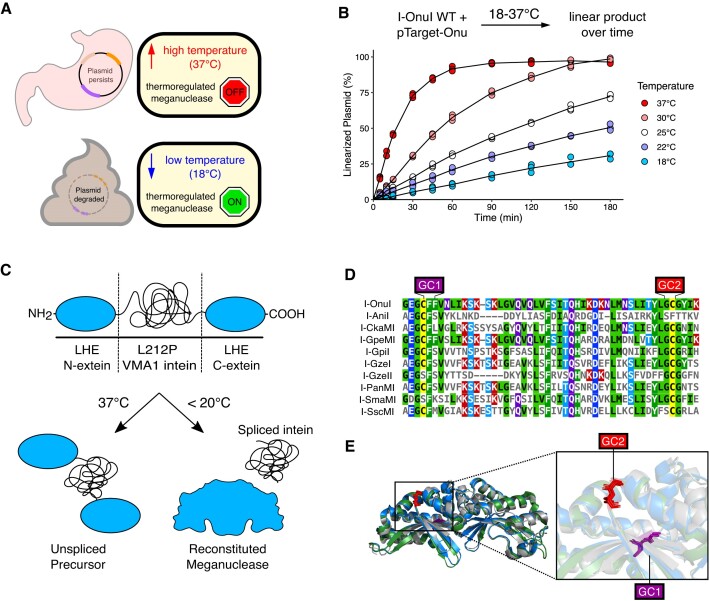
Strategy for the development of a thermosensitive biocontainment endonuclease. (**A**) Schematic of the thermosensitive biocontainment system shown in the context of plasmid containment in the mammalian gut. (**B**) Linear product formation over time *in vitro* for wild-type I-OnuI incubated at different temperatures with pTarget-Onu. The line of best fit through the mean value is plotted for each temperature condition. Each point represents an independent replicate (*n* = 3). (**C**) Thermoregulation of LAGLIDADG homing endonucleases (LHEs) using the L212P VMA1 intein. (top) The VMA1/LHE open reading frame (not to scale). (bottom) A functional reconstituted meganuclease is created by intein splicing at temperatures below 20°C but not at higher temperatures. (**D**) Multiple sequence alignment of 10 LHEs with the two conserved glycine-cysteine sites for the insertion of the VMA1 intein labelled as GC1 and GC2 in purple and red, respectively. (**E**) Crystal structure alignment of the LHEs I-OnuI, I-GpeMI and I-PanMI modified in PyMol. I-OnuI is shown in blue (PDB:6BDA), I-GpeMI in green (PDB:4YHX) and I-PanMI in silver (PDB:5ESP). GC1 and GC2 are highlighted in purple and red, respectively.

Here, we use a different biocontainment approach that is based on temperature as a trigger to post-translationally activate a site-specific DNA endonuclease (Figure 1). Our system is based on the insertion of the temperature-sensitive VMA1 L212P intein into the coding region of LAGLIDADG homing endonucleases (we call these enzymes temperature-sensitive meganucleases, or TSMs) (30–35). The VMA1 L212P intein splices at temperatures below 20°C (33) to activate TSMs for site-specific cleavage, eliminating plasmids carrying the cognate TSM target site from laboratory strains of *Escherichia coli*. TSMs also eliminated plasmids from the probiotic *E. coli* Nissle 1917 strain that had been passaged through the mouse gut when fecal resuspensions were incubated at 18°C but not at 37°C. Our data highlight the utility of a simple, post-translationally activated DNA endonuclease for temperature-regulated biocontainment that is orthogonal to current microbiome editing tools and kill switches, and does not require exogenously added molecules for activation.

## Materials and methods

### Bacterial strains

*E. coli* EPI300 (F′ λ^−^*mcrA* Δ(*mrr-hsdRMS-mcrBC*) ϕ80d*lacZδM15 Δ(lac)X74 recA1 endA1 araD139* Δ(*ara, leu)7697galUgalKrpsL* (Str^*R*^) *nupGtrfAdhfr*) (Epicenter) was used for cloning purposes. *E. coli* NEB5α (*fhuA2Δ(argF-lacZ)U169 phoA glnV44 ϕ80Δ(lacZ)M15 gyrA96 recA1 relA1 endA1 thi-1 hsdR17*) (New England Biolabs) was used for the initial two-plasmid assay. *E. coli* Nissle 1917 (acquired from Dr. Jeremy Burton at Western University) was used in the characterization experiments, escape assays, and mouse experiments. *E. coli* ER2566 (B F^-^, λ^−^*fhuA2*, [*lon*], *ompT lacZ::*T7.1 *gal sulA11* Δ(*mcrC-mrr*) *114*::IS*10* R(*mcr-73*::)miniTN*10*(Tet^S^) *endA1* [*dcm*]) (New England Biolabs) was used for protein expression and purification.

### Plasmid construction

A list of primers is provided in Supplementary Table S1 and plasmids in Supplementary Table S2. All plasmids were assembled in *E. coli* EPI300 using Gibson assembly or Golden Gate Assembly (36,37). All Gibson assemblies used the NEBuilder HiFi DNA Assembly Kit (New England Biolabs). All Golden Gate assemblies and mutagenesis were performed using BsmBI (New England Biolabs). Plasmids were designed in Benchling, gene fragments were ordered through Telesis Bio and assembled on the BioXp™ 3200. Primers were ordered from Integrated DNA Technologies. Wild-type I-OnuI was previously cloned between the NcoI and NotI sites in pProEX-HT-a (Invitrogen and Life Technologies) and pEndo backbone (38,39).

I-OnuI was removed from the original pEndo vector by inverse PCR, using DE-6667 and DE-5792. I-GpeMI and I-PanMI were cloned into the resulting linear plasmid by Gibson assembly. pEndo I-GpeMI was assembled with the linear pEndo amplicon and DE-6369. pEndo I-PanMI was assembled with the linear pEndo amplicon and DE-6377. pEndo I-GpeMI was linearized at GC1 using DE-6291 and DE-6292, and at GC2 using DE-6293 and DE-6294. The VMA1 intein was cloned by Gibson assembly into GC1 of I-GpeMI using DE-6325 and DE-6326, and at GC2 using DE-6327 and DE-6328. pEndo I-PanMI was linearized at GC1 using inverse PCR with primers DE-6307 and DE-6308, and at GC2 using primers DE-6309 and DE-6310. The VMA1 intein was cloned by Gibson assembly into GC1 of I-PanMI using DE-6341 and DE-6342, and at GC2 using DE-6343 and DE-6344. pEndo I-OnuI was linearized at GC1 using inverse PCR with DE-5706 and DE-5707, and at GC2 using DE-5669 and DE-5670. The VMA1 intein was cloned by Gibson assembly into GC1 of I-OnuI using DE-7157 and DE-7158, and at GC2 using DE-5963 and DE-5964. All intein-based plasmids had the L212P mutation introduced using Golden Gate Mutagenesis with DE-6364 and DE-6365 (40). The E22Q and N454Q mutants of the I-OnuI TSM were also created using Golden Gate Mutagenesis using DE-6720 and DE-6721 for E22Q, and DE-6958 and DE-6959 for N454Q at the second glycine-cysteine site. The pBAD promoter and *araC* gene were removed from original iteration of pEndo I-OnuI using DE-5733 and DE-5734. Both primers contained 35-bp overhangs carrying the sequence for the BBaJ23108 Anderson promoter, which was re-circularized by Gibson assembly. pDual was developed from this pEndo variant by opening a site downstream of the I-OnuI TSM ORF using DE-6963 and DE-6964. The I-GpeMI ORF was cloned out of pEndo I-GpeMI using DE-6961 and DE-6962, excluding the BBaJ23108 Anderson promoter. The I-GpeMI ORF was cloned into pEndo with the BBaJ23100 Anderson promoter using DE-6965, DE-6966 and DE-6960.

pTarget was created from a preexisting toxic plasmid (pTox), as previously described (38,41). The *ccdB* DNA gyrase toxin and associated *lac* operon regulatory components were removed from pTox using DE-5737 and DE-5738. Both primers carried 20-bp overhangs that allowed re-circularizing using Gibson assembly. I-GpeMI and I-PanMI target sites were introduced by cloning out the I-OnuI target site using DE-5830 and DE-5831, and new target sites for I-GpeMI and I-PanMI were inserted using DE-6354 and DE-6359, respectively. pTarget-Dual was developed by linearizing pTarget-Onu using inverse PCR with DE-5833 and DE-5834, and introducing an I-GpeMI target site by Gibson assembly using DE-6734. pEndo (I-OnuI TSM), pDual, and pTarget with both I-OnuI and I-GpeMI target sites have been deposited in Addgene (plasmid number 207954, 207884, and 207883). pTarget-gReg was constructed by inserting the CDS of the pGreg-GusR-dCas9.7 plasmid amplified using DE-7400 and DE-7401 into the pTarget plasmid vector. The pTarget vector was linearized at the pGreg CDS insertion site using DE-5833 and DE-5834 and the two linear fragments were assembled by using Gibson assembly.

### *In vitro* cleavage assays

Wild-type I-OnuI was cloned between the NcoI/NotI restriction sites of pProEX-HT-a and over-expressed in *E. coli* ER2566. Cultures were grown to mid-log (OD_600_∼0.5) and protein expression was induced with 1 mM isopropyl-β-d-thiogalactopyranoside (IPTG, BioBasic) overnight at 16°C. Cells were pelleted at 6000 x *g* for 15 min, followed by resuspension in binding buffer (50 mM Tris·HCl pH 8.0, 500 mM NaCl, 1 mM imidazole, 10% glycerol) and lysed via sonication. The cell lysate was centrifuged at 16 000 × *g*. The clarified lysate was loaded onto a 1 mL HisTrap-HP column (Cytiva) and washed with 10 ml binding buffer and 10 ml wash buffer (50 mM Tris·HCl pH 8.0, 500 mM NaCl, 35 mM imidazole, 10% glycerol). I-OnuI was eluted using 5 ml of elution buffer (50 mM Tris·HCl pH 8.0, 500 mM NaCl, 100 mM imidazole, 10% glycerol) and dialyzed against 1 L dialysis buffer (50 mM Tris·HCl pH 8.0, 250 mM NaCl, 30 mM imidazole, 10% glycerol).

Time-point cleavage assays to determine I-OnuI cleavage activity at different temperatures was performed in reaction buffer (50 mM Tris·HCl pH 8.0, 100 mM NaCl, 10 mM MgCl_2_, 1 mM dithiothreitol (DTT)), 400 nM purified I-OnuI, and 10 nM supercoiled target plasmid. Reactions were performed in parallel at 37, 30 25, 22 and 18°C. Reactions were stopped at 10 time points using stop solution (100 mM ethylenediaminetetraacetic acid (EDTA), 160 μg Proteinase K (New England Biolabs), dissolved in Dulbecco’s phosphate-buffered saline (D-PBS)). Cleavage products were visualized on a 1% agarose gel and stained with ethidium bromide. The gel was imaged using the ChemiDoc Imaging System (Bio-Rad) and the proportion of nicked, linear, and supercoiled bands were quantified using the Bio-Rad Image Lab software.

### Bacterial two-plasmid assay

We used a modified version of a two-plasmid assay as previously described (41–44). 50 ng of each pEndo variant was transformed into 50 μl of *E. coli* NEB5α carrying pTarget with the appropriate cognate target sites. Cells were allowed to recover in 1 ml 2xYT media (16 g/l tryptone, 10 g/l yeast extract, 5 g/l NaCl) at 37°C and 225 rpm for 1 hour. Following the initial recovery, cells were added to 1 ml of 2X induction media (2xYT, 200 μg/ml carbenicillin, 0.04% arabinose). The media was then split, and 1 ml was induced at 37°C for 1.5 h and the other 1 ml at 18°C for 2.5 h. After the outgrowth period, dilutions were prepared and spot plated or spread on M9 minimal media agar plates (1× M9 salts, 0.8% wt/vol tryptone, 1% vol/vol glycerol, 1 mM MgSO_4_, 1 mM CaCl_2_, 0.2% wt/vol thiamine, and 0.02% (wt/vol) L-glucose). Plates were incubated overnight at 37°C or 18°C for 5 days. Colonies were counted manually, and plasmid retention was calculated as the ratio of colonies grown on selective M9 media (carbenicillin (100 μg/ml), kanamycin (50 μg/ml)), to non-selective M9 media (kanamycin (50 μg/ml)). A catalytically inactive I-OnuI mutant (E22Q) and a splicing-deficient VMA1 intein mutant (N454Q) were used as negative controls.

### Time-course plasmid survival assay

*E. coli* Nissle 1917 (EcN) carrying pEndo with the I-OnuI TSM, E22Q endonuclease knockout, or N454Q intein splicing knockout were grown overnight at 37°C under selection. Overnight cultures were diluted 1:100 into LB media (100 μg/ml carbenicillin), and incubation continued at 18°C for 12 h. Aliquots of the culture were taken every hour, and plated on non-selective LB agar (100 μg/ml carbenicillin) or selective LB (100 μg/ml carbenicillin, 50 μg/ml kanamycin). Plates were incubated at either 37°C or 18°C, and colonies were counted manually. Plasmid retention was calculated as previously described.

### Quantitative PCR

Experiments were initiated as previously described for the time-course plasmid survival assay. After 2 h of incubation at 18°C, 10 ml of culture was pelleted and resuspended in 500 μl 1× phosphate-buffered saline (PBS). At 12 h this process was repeated, pelleting 500 μl of culture. The resuspensions were boil-lysed at 95°C for 10 min, then immediately stored at -20°C. DNA concentrations were determined using the Qubit 2 fluorometer (Life Technologies), and samples were diluted to 1 ng/μl. Quantitative real-time PCR (qPCR) was performed using SYBR Select Master Mix (Applied Biosystems) on the Viia 7 Real-Time PCR system (ThermoFisher Scientific), amplifying a 150 bp region of the kanamycin resistance gene on pTarget using DE-7269 and DE-7270, and a 150 bp region of the *CspA* gene (ECOLIN_19660) on the chromosome of *E. coli* Nissle 1917 using DE-7271 and DE-7272. Prior to running samples, each primer pair was assessed for off-target activity by gel electrophoresis of the PCR reactions on a 1% agarose gel. Further validation was performed by melt curve analysis after running the qPCR samples. Three biological and five technical replicates were performed for each sample. Each reaction was performed in a total volume of 10 μl, and included 1 ng of DNA and 400 nM of each primer. Thermocycler run parameters were the standard cycling mode, 50°C for 2 min, 95°C for 2 min, followed by 40 cycles at 95°C for 15 seconds and 60°C for 1 minute. Five replicates of a no-template control were also used for each of the primer pairs. Results were analyzed on the QuantStudio Software V1.3 (ThermoFisher Scientific). Data was plotted as the change in the quantity of pTarget relative to *E. coli* Nissle 1917 chromosome copy number. The relative quantity of pTarget and *E. coli* Nissle 1917 gDNA was determined from standard curves produced by serial dilutions of purified pTarget and genomic DNA.

### Bacterial growth curves

EcN carrying the I-OnuI TSM, pEndo E22Q, pEndo N454Q, or the empty pEndo vector were grown overnight at 37°C under selection. The following day, cultures were diluted into 50 ml LB media (100 μg/ml carbenicillin); either 1:100 or 1:20 for 37°C and 18°C growth curves, respectively. Cultures were incubated at either temperature, 225 rpm, until saturated. The OD_600_ was measured every 30 min for 2.5 h for cultures incubated at 37°C, or every hour for 8 h when incubated at 18°C.

### Escape mutant analyses

Escape mutants were obtained by passaging *E. coli* Nissle 1917 carrying pEndo with the I-OnuI TSM, E22Q endonuclease knockout, or N454Q intein splicing knockout at 37°C overnight. All cells also carried the appropriate pTarget plasmids. The following day, each culture was diluted 1:100 into fresh LB and incubated at 18°C for another 12 hrs. Each culture was serially diluted and plated on selective- (100 μg/ml carbenicillin, 50 μg/ml kanamycin) and non-selective (100 μg/ml carbenicillin) LB plates. The escape frequency was calculated as the ratio between the CFU/ml from selective LB incubated at 18°C to the number of colonies from non-selective media. Twenty seven escape mutant colonies were picked from selective LB plates incubated at 18°C, grown overnight, and plasmids extracted using the Monarch Plasmid Miniprep Kit (New England Biolabs). Both pEndo and pTarget were transformed into *E. coli* EPI300 to achieve higher plasmid yield. Plasmids were fully-sequenced using the Nanopore MinION (Oxford Nanopore Technologies) by Flow Genomics.

### β-Glucuronidase activity assays

GusA (β-glucuronidase) units were determined as previously described, with minor modifications (18). Cultures were first grown overnight at 37°C under selection (100 μg/ml carbenicillin, 50 μg/ml kanamycin). The next day, cultures were diluted 1:100 into 50 ml fresh selective LB media, and incubated overnight at 18°C to induce expression of the I-OnuI TSM. On the third day, 500 μl of each culture was pelleted and resuspended in LB only supplemented with carbenicillin. This resuspension was then added to 50 ml fresh LB media (100 μg/ml carbenicillin, 1 mM pNPG (4-nitrophenyl β-D-glucuronide, Sigma-Aldrich)), and incubated at 37°C, 225 rpm for 3 h to induce expression of the glucuronide-regulated plasmid (pTarget-gReg). Following induction, 1 ml of culture was spun down at 13,000 rpm, decanted, and resuspended in 1 ml selective media without pNPG. This washing step was repeated once more. 100 μl of the cell resuspension was transferred to a 96-well plate, and the OD_600_ was measured using a BioTek Epoch 2 Microplate Spectrophotometer. To the remaining cell resuspension, 40 μl of 0.1% SDS (sodium dodecyl sulfate, Fisher Scientific) and 60 μl chloroform (Fisher Scientific) were added. This mixture was vortexed for 30 seconds to permeabilize cells. 20 μl of permeabilized cells were added to 80 μl reaction buffer (150 mM NaCl, 20 mM HEPES, 1.25 mM pNPG, pH 7.5) in a 96-well plate. The absorbance at 410 nm was measured in the BioTek Epoch 2 Microplate Spectrophotometer every minute for 1 h, 37°C, 548 cpm double orbital shaking. GusA units were calculated as previously described (18).

### Mouse experiments

All mouse model experiments used C57BL/6 female mice with three mice per cage. Drinking water and feed were provided *ad libitum*. One day prior to gavage, drinking water was supplemented with ampicillin (1 g/l) and 2.5% sucrose to facilitate knockdown of the gut microbiome. pEndo, pDual, the E22Q endonuclease knockout, and the N454Q intein splicing knockout harbored in *E. coli* Nissle 1917 were grown overnight in selective LB at 37°C. On the day of gavage, the overnight cultures were diluted 1:50 into fresh LB media, and grown to mid-log (OD_600_∼ 0.5). The cells were pelleted and resuspended with 1× PBS, concentrating the cells to 10^8^ CFUs/100 μl. Each mouse was gavaged with 100 μl of the appropriate sample. For three days following gavage, mice fecal pellets were collected daily and resuspended in PBS (150 μl/mg) by vortexing and mechanical agitation. Samples were serially diluted and plated on selective- (100 μg/ml carbenicillin, 50 μg/ml kanamycin) and non-selective (100 μg/ml carbenicillin) MacConkey agar. Colonies were counted manually to determine the CFU/ml for each sample and both temperatures. The escape frequency was calculated as the reduction between the CFU/ml for pEndo and pDual from plates grown at 18°C compared to 37°C.

## Results

### Design of intein-based thermosensitive meganucleases

Our goal was to design a thermoregulated DNA endonuclease activated at low permissive temperatures outside of the mammalian GI tract to promote elimination of recombinant plasmids, but would remain inactive at higher restrictive temperatures while passaging through the GI tract (Figure 1A). We rationalized that after elimination from the human GI tract, bacteria would be exposed to low temperatures characteristic of waste sewage (15°C in the USA (45)) where activation of a biocontainment system would be critical to eliminate recombinant DNA. To this end, we selected LAGLIDADG homing endonucleases (LHEs, also called meganucleases) as candidate kill switches because of their small coding size, long 22-bp target sequences, tolerance to mutational inactivation, and structural conservation (46,47). We first tested the *in vitro* cleavage activity of the well-characterized I-OnuI meganuclease (48) to determine if the enzyme was active at low temperatures (Figure 1B, Supplementary Figure S1). Using a plasmid that contained the I-OnuI cognate target site (pTarget-Onu), we found I-OnuI was active over at least 5 temperatures spanning the 37°C to 18°C range but with a 4-fold reduction in activity at 18°C as compared to 37°C. This data suggests that I-OnuI would be appropriate for a kill switch if the activity could be regulated to low temperatures.

To create a thermoregulated version of I-OnuI, as well as other meganucleases, we re-purposed the *Saccharomyces cerevisiae* vacuolar ATPase (VMA1) L212P intein that splices at or below 20°C to control meganuclease activity (Figure 1C) (32–35). This post-translational activation of a kill switch differs from previous designs that rely on transcriptional control regulated by exogenously added signalling molecules. The VMA1 intein splices most efficiently when inserted downstream of a glycine (G) residue and upstream of a cysteine (C) (49–51), and is enhanced when inserted in a loop region or proximal to the active site of the host protein (52). We found two glycine-cysteine (GC) sites that are conserved amongst 10 aligned meganucleases, with at least one site being found in each meganuclease (Figure 1D). All 10 meganucleases are functionally orthogonal, cleaving different cognate DNA substrates (Supplementary Figure S2). I-OnuI, I-GpeMI, and I-PanMI possessed both of the GC sites and aligning the crystal structures revealed that the GC sites are located in the C-terminal domain of each enzyme. The first GC site (GC1) is present in a β-sheet that forms part of the I-OnuI protein-DNA interface while the second GC site (GC2) is present in an exposed alpha helix (Figure 1E). We independently inserted the temperature sensitive L212P (TS) and wild-type (WT) VMA1 inteins into I-OnuI, I-GpeMI and I-PanMI (Supplementary Figure S3). Hereafter, the meganuclease constructs with the L212P temperature-sensitive intein insertion are referred to as TSMs (thermosensitive meganucleases).

### Testing the thermosensitivity of TSMs

**Figure 2.**
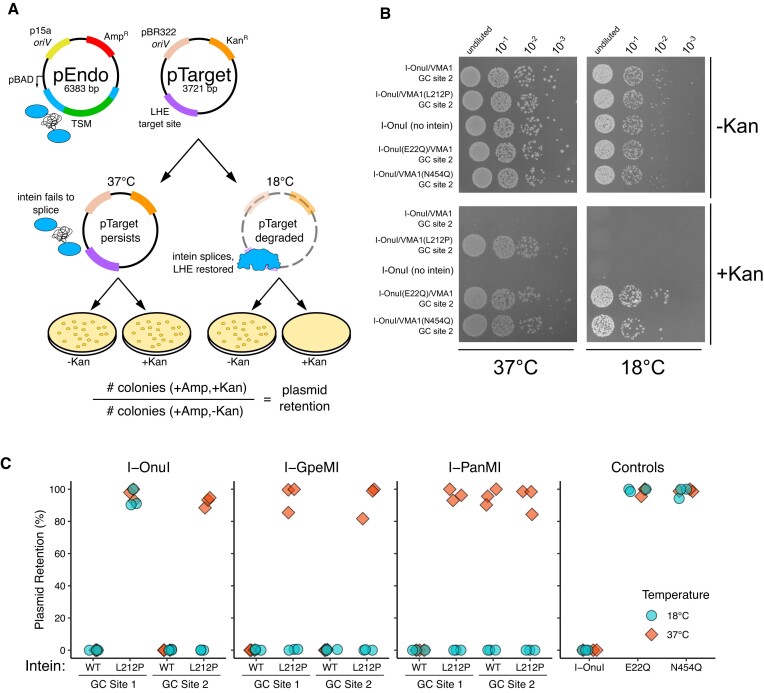
Thermoregulation of endonuclease activity using an intein-based approach. (**A**) Schematic of pEndo and pTarget used in the two-plasmid selection experiments. *oriV*, plasmid origin of replication; pBAD, arabinose-inducible promoter; TSM, temperature-sensitive meganuclease; Amp^R^, ampicillin resistance gene; Kan^R^, kanamycin resistance gene. The LHE target site is cleaved by the spliced TSM at the permissive temperature and the extent of pTarget loss can be determined by the ratio of colonies on solid media containing (+Kan) or lacking (-Kan) kanamycin. (**B**) I-OnuI TSM activity in *E. coli* NEB5*a* on M9 minimal media incubated at either 37°C or 18°C and spot plated on solid media containing (+Kan) or lacking (-Kan) kanamycin. (**C**) Two-plasmid assay in *E. coli* NEB5*a* with TSMs developed from three LHEs. Plasmid retention was calculated as the ratio of colonies grown on M9 minimal media with ampicillin and kanamycin, over media with only ampicillin. Each data point represents individual replicates (n=3).

We next designed an *E. coli* two-plasmid assay to assess the thermosensitivity of meganucleases and TSMs. The assay included pEndo that expressed either the meganuclease or TSM from an arabinose-regulated promoter (53) and pTarget that contained the meganuclease target site. Active meganucleases/TSMs will cleave their cognate target sites on pTarget to promote elimination of pTarget by the RecBCD pathway (54) and subsequent loss of kanamycin resistance, with the TSMs only being active at low temperatures (Figure 2A).

We first cloned the I-OnuI GC2 TSM into pEndo and assessed elimination of pTarget-Onu by spot plating over 1000-fold dilutions at 18°C and 37°C (Figure 2B). Under selective conditions to assess pTarget-Onu loss, we observed no growth at 18°C and robust growth at 37°C (Figure 2B, +Kan condition). We also determined pTarget-Onu retention by the ratio of colony counts on media containing or lacking kanamycin and observed no pTarget-Onu retention at 18°C as compared to near 100% retention at 37°C (Figure 2C). These data are consistent with the L212P intein splicing at 18°C to produce a functional I-OnuI meganuclease that promotes elimination of pTarget and subsequent sensitivity to kanamycin. To confirm these observations, we performed parallel experiments with the WT VMA1 intein inserted in I-OnuI GC2, finding no growth at either 18°C or 37°C, indicating that splicing at both temperatures created functional I-OnuI to eliminate pTarget (Figure 2B and C). The wild-type I-OnuI meganuclease with no intein insertion promoted pTarget-Onu elimination at either 18°C and 37°C (Figure 2b and c). We also created endonuclease dead (E22Q) and intein-splicing dead (N454Q) variants in the I-OnuI GC2 construct. These variants promoted growth at either temperature, demonstrating that pTarget elimination is dependent on both endonuclease activity and intein splicing (Figure 2B and C). Intein splicing did not create a toxic meganuclease at either temperature as we observed robust growth on plates lacking kanamycin.

To extend these findings to the second GC site in I-OnuI, and to other meganucleases, we created versions of pEndo carrying meganuclease variants as well as pTarget variants with the cognate target sites for each meganuclease. As shown in Figure 2C, the insertion of the L212P VMA1 intein into the GC1 and GC2 sites of I-PanMI and I-GpeMI generated TSMs, whereas only the I-OnuI GC2 site generated a TSM. Interestingly, inserting the wild-type VMA1 intein into GC2 in I-PanMI generated a thermosensitive phenotype. As seen for the spot plating data (Figure 2B), we observed near 100% plasmid retention for variants with substitutions that inactivated I-OnuI endonuclease activity (E22Q) and intein splicing (N454Q) (Figure 2C). Orthogonality of the system was demonstrated by co-transforming pEndo programmed with the I-OnuI TSM and pTarget carrying the I-GpeMI site (pTarget-Gpe); pTarget-Gpe retention was ∼100% at 18°C, indicating lack of cleavage by I-OnuI (Supplementary Figure S4). Collectively, these experiments demonstrated that a set of orthogonal temperature-sensitive meganucleases (TSMs) were created by the insertion of the L212P VMA1 intein variant at splicing-permissive GC sites in I-OnuI, I-PanMI and I-GpeMI.

### Kinetics of TSM activation

**Figure 3.**
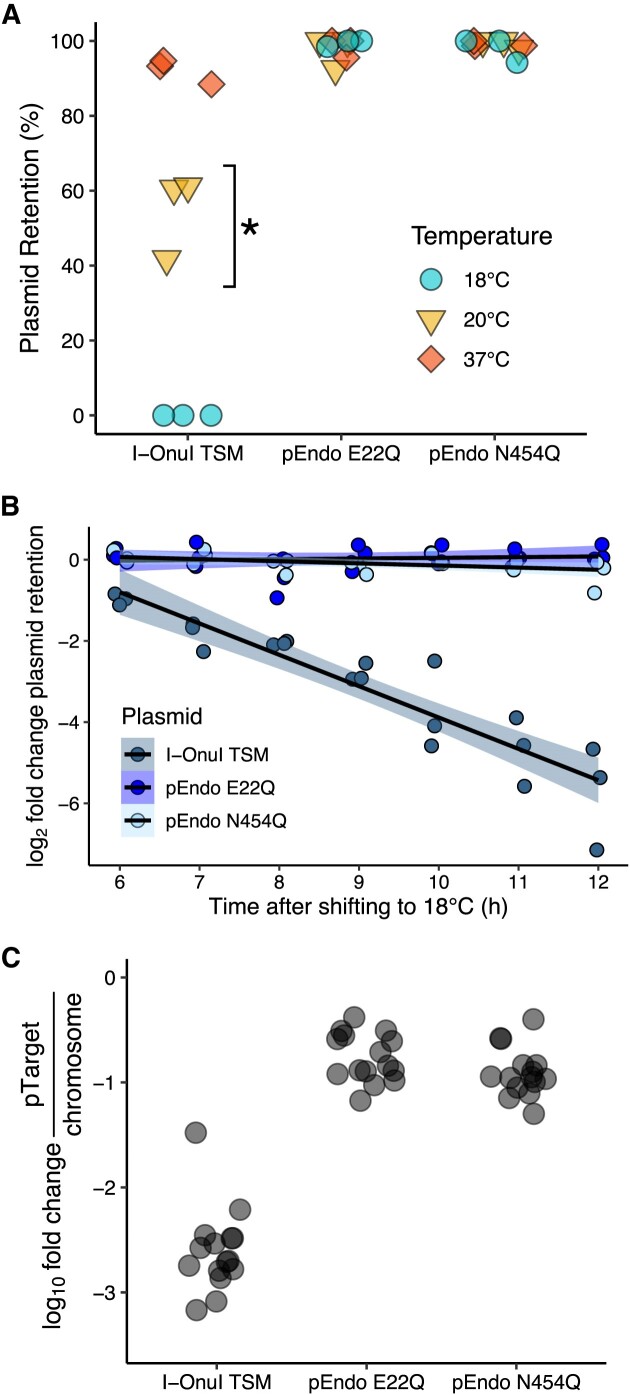
Thermosensitivity of the I-OnuI TSM in *E. coli* Nissle 1917 (EcN). (**A**) Two-plasmid assay with the I-OnuI TSM to determine the temperature at which plasmid cleavage is no longer observed. Each data point represents individual replicates (n=3). The asterisk (*) indicates small colony morphology observed on solid LB media incubated at 20°C. (**B**) Elimination of pTarget-Onu *in vivo* in liquid culture over time. Aliquots of the liquid culture were taken at the specified time points and plated on solid LB media, Plasmid retention was calculated at the indicated time points as described in Figure 2C for the indicated variants. Each data point represents individual replicates (*n* = 3). (**C**) Change in the quantity of pTarget-Onu relative to the *cspA* gene on the EcN chromosome number after 12 h of incubation at 18°C determined by quantitative PCR. Three biological and five technical replicates were performed for each sample, and each data point represents one of the replicates.

We next sought to characterize the I-OnuI GC2 TSM by determining the kinetics at the optimal restrictive temperature. We found that the I-OnuI TSM was fully functional at 18°C, partially functional at 20°C and exhibited a small-colony phenotype on solid media plates, and was inactive at higher temperatures (Figure 3A). The small-colony phenotype was likely the result of incomplete pTarget-Onu cleavage, resulting in partial sensitivity to kanamycin. Further profiling was performed at 18°C.

We next profiled the *in vivo* kinetics of TSM activation by diluting saturated cultures containing I-OnuI TSM and pTarget-Onu into fresh media lacking kanamycin and incubating the cultures at 18°C for up to 12 h. At every hour, an aliquot was plated on solid media with or without kanamycin to calculate pTarget-Onu depletion. For these experiments, we used the probiotic strain *E. coli* Nissle 1917 (EcN) that has GRAS (generally recognized as safe) status instead of the NEB5α strain (55–58). We also removed the pBAD regulatory cassette from pEndo, replacing it with the weak, constitutively active Anderson promoter (BBaJ23108) in anticipation of limiting complications of using sugar-related promoters for *in vivo* mouse experiments. With the I-OnuI TSM, minimal pTarget-Onu depletion was observed over the first six h. In contrast, from 6-12 h after shifting to 18°C, pTarget-Onu was eliminated at a linear rate (Figure 3B). We observed no measurable pTarget-Onu elimination with cultures containing the pEndo N454Q intein splicing mutant or the I-OnuI E22Q endonuclease dead mutant (Figure 3B). We estimate that by the end of the 12-h incubation period, the I-OnuI TSM kill-switch had a 50-fold rate of pTarget-Onu depletion over both the negative controls. To determine if constitutive endonuclease expression induces cytotoxicity, we performed growth-curve assays at both 37 and 18°C (Supplementary Figure S5). We found no measurable difference in the rate of growth at either temperature between strains with active or non-functional TSMs, or with strains that do not express an endonuclease.

To provide an independent measure of the I-OnuI TSM activity, we performed quantitative PCR on total DNA isolated after 12 h at 18°C to determine the change in pTarget-Onu abundance relative to the *cspA* gene on the chromosome. The I-OnuI TSM reduced pTarget-Onu abundance ∼1000-fold *in vivo* as compared to EcN with the I-OnuI E22Q endonuclease dead and VMA1 N45Q splicing dead constructs (Figure 3C and Figure S6). Taken together, these data show that I-OnuI TSM is active at 18°C but that at least 6 h incubation *in vivo* is required before clearance of pTarget-Onu can be observed.

### Multiplexing TSMs reduces the frequency of escape mutants

**Figure 4.**
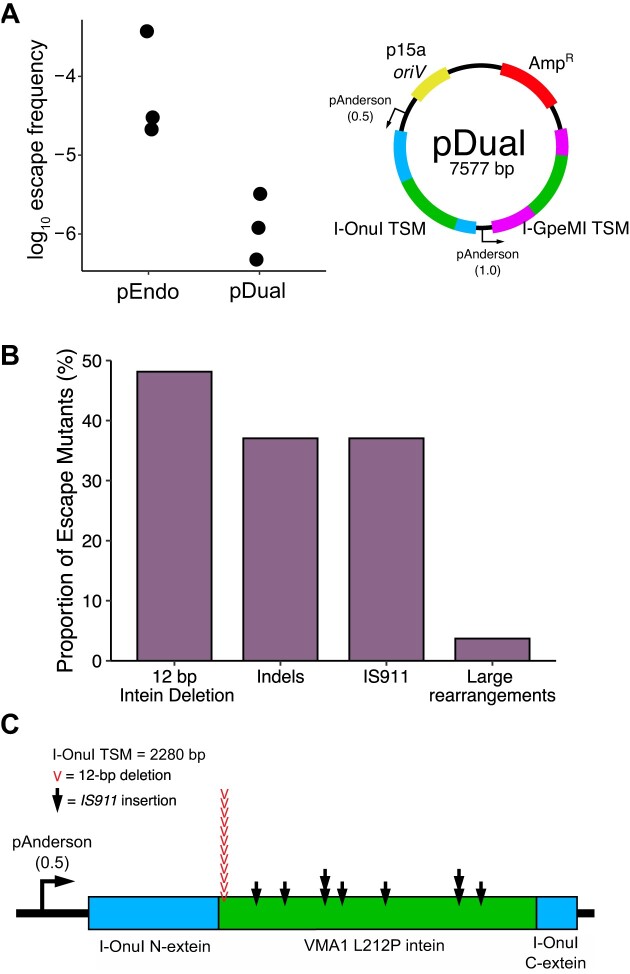
Characterizing escape and inactivation of the I-OnuI TSM. (**A**) Escape frequency from a single- (pEndo) and dual-endonuclease (pDual) setup. (left) The frequency of TSM inactivation in pEndo and pDual. The escape frequency was determined by the number of colonies grown on solid LB media with kanamycin relative to media without kanamycin. Each data point represents individual replicates (*n* = 3). (right) Schematic of the dual-endonuclease plasmid, pDual. *oriV*, plasmid origin of replication; Amp^R^, ampicillin resistance gene; pAnderson, constitutively active Anderson promoter; TSM, temperature-sensitive meganuclease. Two variations of the Anderson promoter were used, with either high- (1.0) or mid- (0.5) expression relative to the cognate Anderson promoter. Each Anderson promoter controls the expression of a separate TSM. (**B**) Identification of inactivating mutations in the TSM coding region from escape mutants of pEndo in *E. coli* Nissle 1917. Data is shown as a bar plot, with the percentage of each of the type of mutations shown relative to the total number of escape plasmids. (**C**) Mapping the locations of IS*911* insertions and the 12 bp VMA1 intein deletion on the I-OnuI TSM. Black arrows represent IS*911* insertion sites on the I-OnuI TSM open reading frame, and red arrows represent the location of the 12 bp VMA1 intein deletion.

An important characteristic of a biocontainment system is the rate of escape. In our system, escape would be characterized by the appearance of colonies on solid media with kanamycin, as pTarget was not eliminated. We determined the escape frequency for the I-OnuI TSM to be between 10^−4^ and 10^−5^ (Figure 4A), similar to reported frequencies for other endonuclease-based kill switches which initiate killing at a single target site, albeit prior to further optimization (59). To understand how the TSM was being inactivated, we isolated total plasmid DNA from 27 escapees and used Oxford Nanopore sequencing to identify mutations in both pEndo and pTarget. A significant fraction of the escape mutants possessed large insertions in pEndo, many of which created frame-shift mutations resulting in truncated endonucleases (Figure 4B, Supplementary Figure S7). Interestingly, 10 of these insertions produced a consistent size increase in pEndo from the original 5.2 kb plasmid to a ∼6.7 kb plasmid and all insertions were in the VMA1 coding region (Figure 4C). We identified the insertions as having similarity with IS*911*, a member of the IS*3* bacterial insertion sequence family (60). IS*911* is known to be temperature-sensitive (61), increasing in both transposition frequency and production of IS*911*-associated proteins as temperature decreases, suggesting that many of these insertions likely occurred upon shifting the incubation temperature to 18°C. We also identified a hotspot for a common 12-bp deletion in the VMA1 intein sequence that was found in 13 of the escape mutants (Figure 4B and C). This deletion may occur more frequently in this region due to a 9-bp palindromic region found on either side of the 12-bp VMA1 deletion (Supplementary Figure S8), contributing to genetic instability at this locus (62–65).

The above analysis revealed that escape mutants arose from mutations in the TSM coding region, suggesting that multiplexing TSMs on pEndo could decrease the escape frequency. We constructed a pEndo variant expressing two TSMs by placing the coding region of I-GpeMI with the L212P VMA1 intein inserted at GC2 (I-GpeMI TSM) downstream of the I-OnuI coding region (Figure 4A). To avoid recombination between genetic elements on pEndo, we used a different variant of the Anderson promoter (BBaJ23100) to express the I-GpeMI TSM and modified the DNA sequence of the L212P VMA1 intein within I-GpeMI, creating a synonymous substitution every third codon. An I-GpeMI target site was also inserted into pTarget. With pDual, we found an ∼10-fold decrease in the escape frequency relative to pEndo (Figure 4A). This result demonstrates that multiplexing orthogonal TSMs to introduce redundancy can reduce the escape frequency.

### TSMs function orthogonally to CRISPR-based regulatory modules

**Figure 5.**
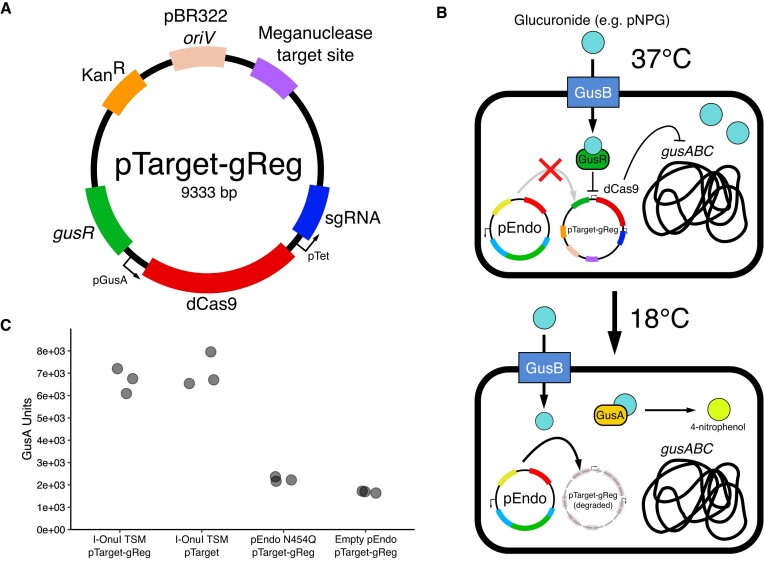
Containment of a biotherapeutic plasmid using the I-OnuI TSM. (**A**) Schematic of the pTarget-gReg plasmid. *oriV*, plasmid origin of replication; Kan^R^, kanamycin resistance gene; *gus^R^*, glucuronide-responsive transcription factor; pGusA, promoter from the *gusA* regulatory region; dCas9, catalytically-inactive Cas9; pTet, constitutively-active tetracycline resistance gene promoter; sgRNA, single guide RNA. (**B**) Schematic outlining the β-glucuronidase activity assay. Cleavage and subsequent degradation of pTarget-gReg relieves suppression of the endogenous *gus* operon, restoring GusA activity to wild-type levels. GusA activity is reported by the formation of the chromogenic 4-nitrophenol product by cleavage of pNPG (4-nitrophenyl-β-D-glucuronide). (**C**) β-Glucuronidase activity assay in *E. coli* Nissle 1917 with the I-OnuI TSM. Shown is GusA activity in strains with active nuclease (I-OnuI TSM), no target site (pTarget), I-OnuI TSM with an intein-splicing mutations (pEndo N454Q), or backbone vector (pEndo). Each data point represents an individual replicates (*n* = 3).

Our lab previously developed a biotherapeutic plasmid (pGreg) where dCas9 is regulated by the GusR transcription factor that responds to exogenous glucuronides to repress GusA expression and alleviate the toxic effects of re-activation of glucuronidated xenobiotic compounds by microbial β-glucuronidase (18). We sought to determine if the I-OnuI TSM was compatible with pGreg by constructing pTarget-gReg which incorporates the GusR-dCas9-guideRNA regulatory module into pTarget (Figure 5A). At 37°C, pTarget-gReg should remain stable in cells also harbouring the I-OnuI TSM (pEndo-Onu), resulting in dCas9-mediated repression of the *gusA* β-glucuronidase when incubated with glucuronides. However, at 18°C the degradation of pTarget-gReg by the I-OnuI TSM should alleviate repression of *gusA* and result in increasing amounts of 4-nitrophenol, produced when pNPG is cleaved by GusA (Figure 5B). As shown in (Figure 5C, Supplementary Figure S9) GusA activity is not downregulated when EcN carrying pTarget-gReg and pEndo with an active TSM is incubated at 18°C with pNPG supplementation. In contrast, cell carrying pEndo with the intein-dead N454Q mutant or without a TSM coding region displayed reduced GusA activity, consistent with dCas9 downregulation of *gusA* expression. These data shown that the TSM biocontainment system does not interfere with CRISPR-based regulatory modules and can function to restrict the activity of biotherapuetic plasmids at the permissive temperature.

### TSM restricts plasmids to the mouse GI tract

**Figure 6.**
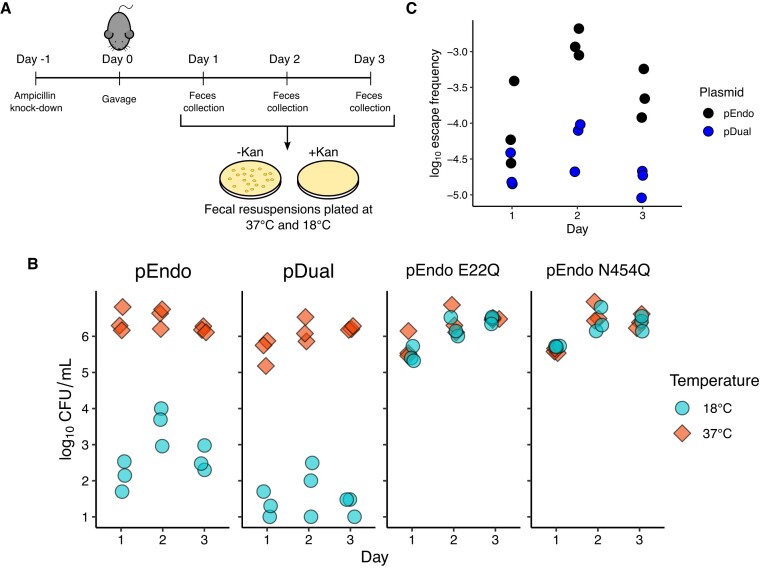
Containment of plasmids with single or multiplexed TSMs in the mouse gut. (**A**) Schematic outlining the mouse model experiments. Gut microbiome knockdown was performed with 1 g/l ampicillin and 2.5% sucrose. Mice were gavaged with 10^8^ CFUs of pEndo or pDual. Fecal samples were resuspended in PBS (150μl/mg) and plated on solid media containing (+Kan) or lacking (–Kan) kanamycin to determine pTarget loss. (**B**) Depletion of pTarget by pEndo and pDual. Plasmid retention was calculated as the ratio of colonies grown on MacConkey agar as depicted in panel (**A**). Each data point represents fecal samples collected from the 3 mice used in each experimental group (*n* = 3). (**C**) Escape frequency of pEndo and pDual in a mouse model. The escape frequency was determined by the ratio of colonies recovered from plates incubated at 18°C relative to colonies recovered at 37°C. Each data point represents an individual replicate (*n* = 3).

To demonstrate that the I-OnuI and I-GpeMI TSM kill-switches can restrict plasmids to the gut environment, we independently passaged EcN harbouring pEndo or pDual and the appropriate pTarget vector through C57BL/6 mice, collecting fecal samples over three days (Figure 6A). Both of the EcN strains harbouring active TSMs demonstrated a 10^3^ to 10^5^ fold reduction in pTarget retention when fecal resuspensions were incubated at 18°C, observed as a decrease in recovered CFU/ml on kanamycin-supplemented media (Figure 6B). No significant reduction in recovered CFU/ml was observed with fecal suspensions from TSM-active strains incubated at 37°C as compared to the E22Q nuclease dead or N454Q intein splicing inactive controls. We found that the escape frequency of each of the pEndo or pDual TSM strains was ∼10-fold higher *in vivo* compared to frequencies found for *in vitro* experiments (compare Figure 6A with B). However, we noted that pDual maintained the 10-fold reduction in escape frequency as compared to pEndo, suggesting that multiplexing orthogonal TSMs is broadly applicable to different environments. Taken together, this data confirms that the TSM is inactive while in the mouse gut but is activated outside of the mouse gut at 18°C. Furthermore, no side effects were observed in any of the mice, indicating that the circuit appears stable and safe for at least three days in the mouse gut.

## Discussion

We created a series of thermally-regulated site-specific meganucleases controlled by intein splicing to promote degradation and biocontainment of DNA at low temperatures outside of the mammalian gut, or for other applications where a suitable temperature gradient exists. In contrast to past kill-switches that are primarily regulated by transcriptional mechanisms, our system functions at the post-translational level and is controlled by the temperature-sensitive VMA1 L212P intein that, in the context of meganucleases, splices at 20°C or below. The high structural conservation between meganuclease family members suggests that TSMs could be created beyond those tested here by inserting the VMA1 L212P intein into the GC1 and GC2 sites. VMA1 inteins (or other inteins) with different splicing permissive temperatures could be inserted into meganucleases to fine-tune endonuclease activity to a desired temperature depending on the application or organism. This strategy could also be used to create different temperature-controlled site-specific endonucleases, such as the GIY-YIG family endonuclease I-TevI that is permissive to intein insertion (66), or other toxin systems that promote DNA degradation and bacterial killing.

The small coding size of meganucleases (under 1-kb) and lack of elaborate transcriptional circuits to control gene expression simplifies both plasmid construction and multiplexing of different TSMs. Our biocontainment system does not require engineering of the bacterial genome, or require exogenously added signalling molecules to repress or induce TSM expression. This simplicity confers a number of advantanges, including lack of regulatory cross-talk that would facilitate multiplexing of TSMs with other biocontainment systems such as temperature-sensitive CRISPR nucleases. From a practical viewpoint, the TSM biocontainment system is well suited to experiments that introduce engineered microbes carrying recombinant plasmids into the mammalian GI tract simply because eliminated feces are immediately introduced to a permissive temperature for intein splicing, TSM activation and plasmid degradation (the mean temperature of sewage in the United States is 15°C). We can envision other applications for TSMs, including the elimination of bacterial strains that are engineered to carry TSM target sites, the curing of plasmids from bacteria, yeast or other species that can grow at a wide range of temperatures, or industrial applications where plasmid-containing cultures can be shifted to low temperatures to induce plasmid destruction. Lastly, layering of TSMs with functionally distinct kill switches could achieve a level of degradation of plasmid or genomic DNA to satisfy biocontainment concerns in therapeutic applications.

## Supplementary Material

gkad1247_Supplemental_Files

## Data Availability

The original data generated in this study is available as a supplementary file available at NAR online. The TSM plasmids and target plasmids created in this study have been deposited in Addgene (207883, 207884 and 207954).
