# Supplementary material for: Intein-based thermoregulated meganucleases for containment of genetic material
Source: Nucleic Acids Res. 2024 Jan 5;52(4):2066–77. doi: 10.1093/nar/gkad1247 (PMC10899782; doi:10.1093/nar/gkad1247)
Supplement: gkad1247_Supplemental_Files [file gkad1247_supplemental_files.zip › Foo_supplemental_figures_revised.pdf]

Supplemental Data for:

Intein-based thermoregulated meganucleases for biocontainment of genetic material

Gary W. Foo[1], Christopher D. Leichthammer[1], Ibrahim M. Saita[1], Nicholas D. Lukas[1], Izabela Z. Batko[2], David E. Heinrichs[2] and David R. Edgell[1*]

[1]Department of Biochemistry, Schulich School of Medicine and Dentistry, London, Ontario N6A 5C1, Canada
[2]Department of Microbiology and Immunology, Schulich School of Medicine and Dentistry, London, Ontario N6A5C1, Canada

This document includes:
Figs. S1-S9

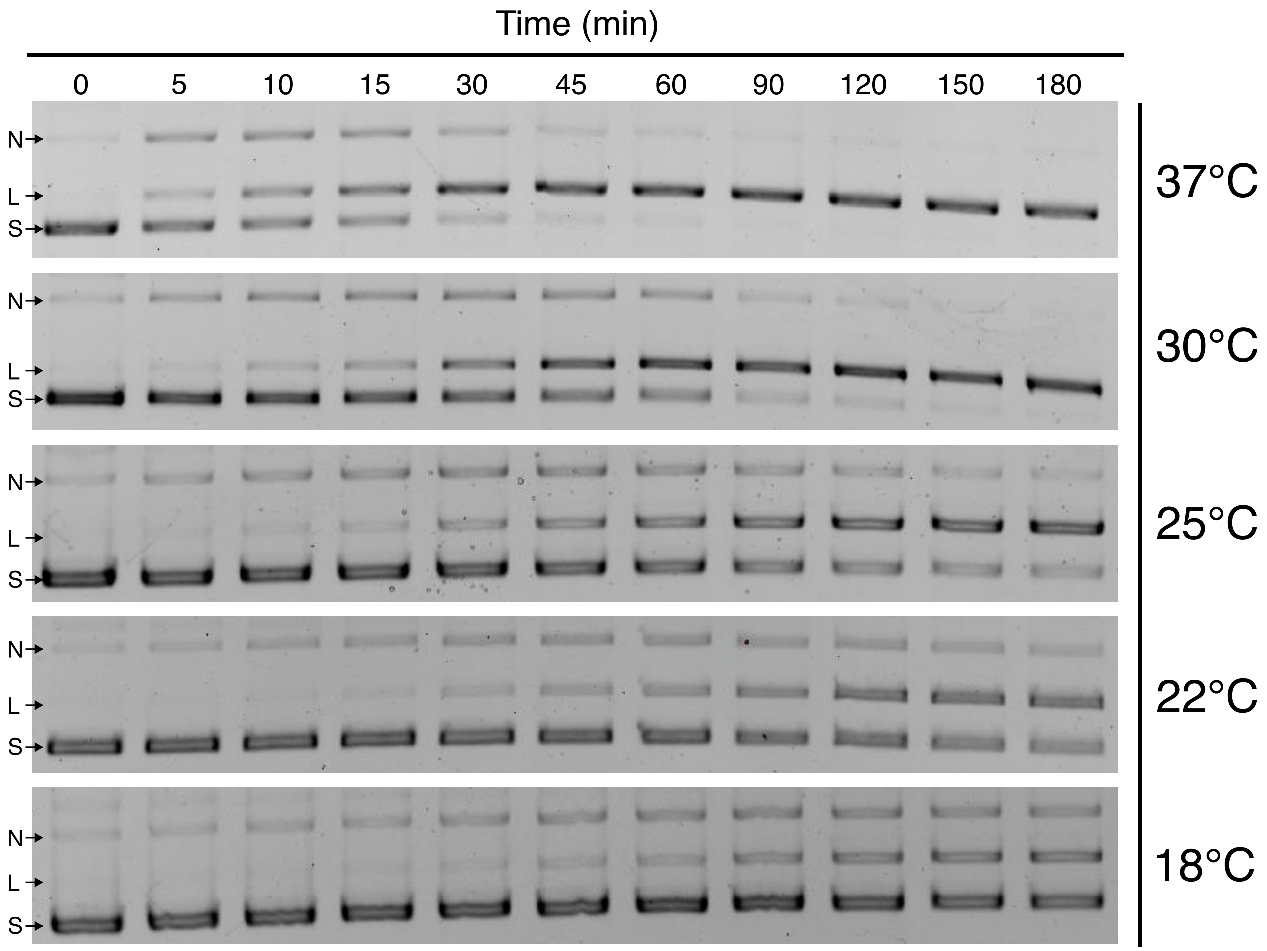

Figure S1. *in vitro* cleavage activity of wild-type I-OnuI against supercoiled plasmid incubated different temperatures. Labeled are the nicked (N), linear (L), and supercoiled (S) bands on each gel. Images cropped for publication.

I-OnuI	TTTCCACTTATTCAACCTTTTA
I-AniI	CTGAGGAGGTTTCTCTGTAAAG
I-CkaMI	AAGTACCCTTTAAACCTAATTA
I-GpeMI	TTTCCGCTTATTCAACCCTTTA
I-GpiI	TTTTCCTGTATATGACTTAAAT
I-GzeI	GCCCCTCATAACCCGTATCAAG
I-GzeII	ATGGGTACCATATTGGTACAAA
I-PanMI	GCTCCTCATAATCCTTATCAAG
I-SmaMI	TATCCTCCATTATCAGGTGTAC
I-SscMI	AGGTACCCTTTAAACCTATTAA

Figure S2. Cognate DNA substrates for 10 LAGLIDADG homing endonucleases. Highlighted in red are the “critical four” residues, for which nucleotide substitutions are least tolerated.

>VMA1
CFAKGTNVLMADGSIECIENIEVGNKVMGKDGRPREVIKLPRGRETMYSVVQKSQHRA
HKSDSSREVPELLKFTCNATHELVVRTPRSVRRLSRTIKGVEYFEVITFEMGQKKAPDG
RIVELVKEVSKSYPISEGPERANELVESYRKASNKAYFEWTIEARDLSLLGSHVRKATYQ
TYAPILYENDHFFDYMQKSKFHLTIEGPKVLAYLLGLWIGDGLSDRATFSVDSRDTSLME
RVTEYAEKLNLCAEYKDRKEPQVAKTVNLYSKVVRGNGIRNNLNTENPLWDAIVGLGFL
KDGVKNIPSFLSTDNIGTRETFLAGLIDSDGYVTDEHGIKATIKTIHTSVRDGLVSLARSL
GLVVSVNAEPAKVDMNGTKHKISYAIYMSGGDVLLNVLSKCAGSKKFRPAPAAAFAREC
RGFYFELQELKEDDYYGITLSDDSDHQFLLANQVVVHN

>WT I-OnuI
MGSAYMSRRESINPWILTGFADAEGSFLLRIRNNNKSSVGYSTELGFQITLHNKDKSILE
NIQSTWKVGVIANSGDNAVSLKVTRFEDLKVIIDHFEKYPLITQKLGDYMLFKQAFCVME
NKEHLKINGIKELVRIKAKLNWGLTDELKKAFPEIISKERSLINKNIPNFKWLAGFTSGEG
CFFVNLIKSKSKLGVQVQLVFSITQHIKDKNLMNSLITYLGCGYIKEKNKSEFSWLDFVV
TKFSDINDKIIPVFQENTLIGVKLEDFEDWCKVAKLIEEKKHLTESGLDEIKKIKLNMNKG
RVF*

>I-OnuI/VMA1 (GC1)
MGSAYMSRRESINQWILTGFADAEGSFLLRIRNNNKSSVGYSTELGFQITLHNKDKSILE
NIQSTWKVGVIANSGDNAVSLKVTRFEDLKVIIDHFEKYPLITQKLGDYMLFKQAFCVME
NKEHLKINGIKELVRIKAKLNWGLTDELKKAFPEIISKERSLINKNIPNFKWLAGFTSGEG
CFAKGTNVLMADGSIECIENIEVGNKVMGKDGRPREVIKLPRGRETMYSVVQKSQHRA
HKSDSSREVPELLKFTCNATHELVVRTPRSVRRLSRTIKGVEYFEVITFEMGQKKAPDG
RIVELVKEVSKSYPISEGPERANELVESYRKASNKAYFEWTIEARDLSLLGSHVRKATYQ
TYAPILYENDHFFDYMQKSKFHLTIEGPKVLAYLLGLWIGDGLSDRATFSVDSRDTSLME
RVTEYAEKLNLCAEYKDRKEPQVAKTVNLYSKVVRGNGIRNNLNTENPLWDAIVGLGFL
KDGVKNIPSFLSTDNIGTRETFLAGLIDSDGYVTDEHGIKATIKTIHTSVRDGLVSLARSL
GLVVSVNAEPAKVDMNGTKHKISYAIYMSGGDVLLNVLSKCAGSKKFRPAPAAAFAREC
RGFYFELQELKEDDYYGITLSDDSDHQFLLANQVVVHNCFFVNLIKSKSKLGVQVQLVF
SITQHIKDKNLMNSLITYLGCGYIKEKNKSEFSWLDFVVTKFSDINDKIIPVFQENTLIGVK
LEDFEDWCKVAKLIEEKKHLTESGLDEIKKIKLNMNKGRVF*

>I-OnuI/VMA1 (GC2)
MGSAYMSRRESINQWILTGFADAEGSFLLRIRNNNKSSVGYSTELGFQITLHNKDKSILE
NIQSTWKVGVIANSGDNAVSLKVTRFEDLKVIIDHFEKYPLITQKLGDYMLFKQAFCVME
NKEHLKINGIKELVRIKAKLNWGLTDELKKAFPEIISKERSLINKNIPNFKWLAGFTSGEG
CFFVNLIKSKSKLGVQVQLVFSITQHIKDKNLMNSLITYLGCFAKGTNVLMADGSIECIEN
IEVGNKVMGKDGRPREVIKLPRGRETMYSVVQKSQHRAHKSDSSREVPELLKFTCNAT
HELVVRTPRSVRRLSRTIKGVEYFEVITFEMGQKKAPDGRIVELVKEVSKSYPISEGPER
ANELVESYRKASNKAYFEWTIEARDLSLLGSHVRKATYQTYAPILYENDHFFDYMQKSK
FHLTIEGPKVLAYLLGLWIGDGLSDRATFSVDSRDTSLMERVTEYAEKLNLCAEYKDRK
EPQVAKTVNLYSKVVRGNGIRNNLNTENPLWDAIVGLGFLKDGVKNIPSFLSTDNIGTR
ETFLAGLIDSDGYVTDEHGIKATIKTIHTSVRDGLVSLARSLGLVVSVNAEPAKVDMNGT
KHKISYAIYMSGGDVLLNVLSKCAGSKKFRPAPAAAFARECRGFYFELQELKEDDYYGI
TLSDDSDHQFLLANQVVVHNCGYIKEKNKSEFSWLDFVVTKFSDINDKIIPVFQENTLIG
VKLEDFEDWCKVAKLIEEKKHLTESGLDEIKKIKLNMNKGRVF*

>I-OnuI(E22Q)/VMA1 (GC2)
MGSAYMSRRESINQWILTGFADAQGSFLLRIRNNNKSSVGYSTELGFQITLHNKDKSIL
ENIQSTWKVGVIANSGDNAVSLKVTRFEDLKVIIDHFEKYPLITQKLGDYMLFKQAFCVM
ENKEHLKINGIKELVRIKAKLNWGLTDELKKAFPEIISKERSLINKNIPNFKWLAGFTSGE
GCFFVNLIKSKSKLGVQVQLVFSITQHIKDKNLMNSLITYLGCFAKGTNVLMADGSIECIE
NIEVGNKVMGKDGRPREVIKLPRGRETMYSVVQKSQHRAHKSDSSREVPELLKFTCN
ATHELVVRTPRSVRRLSRTIKGVEYFEVITFEMGQKKAPDGRIVELVKEVSKSYPISEGP
ERANELVESYRKASNKAYFEWTIEARDLSLLGSHVRKATYQTYAPILYENDHFFDYMQK
SKFHLTIEGPKVLAYLLGLWIGDGLSDRATFSVDSRDTSLMERVTEYAEKLNLCAEYKD
RKEPQVAKTVNLYSKVVRGNGIRNNLNTENPLWDAIVGLGFLKDGVKNIPSFLSTDNIG
TRETFLAGLIDSDGYVTDEHGIKATIKTIHTSVRDGLVSLARSLGLVVSVNAEPAKVDMN
GTKHKISYAIYMSGGDVLLNVLSKCAGSKKFRPAPAAAFARECRGFYFELQELKEDDYY
GITLSDDSDHQFLLANQVVVHNCGYIKEKNKSEFSWLDFVVTKFSDINDKIIPVFQENTLI
GVKLEDFEDWCKVAKLIEEKKHLTESGLDEIKKIKLNMNKGRVF*

>I-OnuI/VMA1(N454Q) (GC2)
MGSAYMSRRESINQWILTGFADAEGSFLLRIRNNNKSSVGYSTELGFQITLHNKDKSILE
NIQSTWKVGVIANSGDNAVSLKVTRFEDLKVIIDHFEKYPLITQKLGDYMLFKQAFCVME
NKEHLKINGIKELVRIKAKLNWGLTDELKKAFPEIISKERSLINKNIPNFKWLAGFTSGEG
CFFVNLIKSKSKLGVQVQLVFSITQHIKDKNLMNSLITYLGCFAKGTNVLMADGSIECIEN
IEVGNKVMGKDGRPREVIKLPRGRETMYSVVQKSQHRAHKSDSSREVPELLKFTCNAT
HELVVRTPRSVRRLSRTIKGVEYFEVITFEMGQKKAPDGRIVELVKEVSKSYPISEGPER
ANELVESYRKASNKAYFEWTIEARDLSLLGSHVRKATYQTYAPILYENDHFFDYMQKSK
FHLTIEGPKVLAYLLGLWIGDGLSDRATFSVDSRDTSLMERVTEYAEKLNLCAEYKDRK
EPQVAKTVNLYSKVVRGNGIRNNLNTENPLWDAIVGLGFLKDGVKNIPSFLSTDNIGTR
ETFLAGLIDSDGYVTDEHGIKATIKTIHTSVRDGLVSLARSLGLVVSVNAEPAKVDMNGT
KHKISYAIYMSGGDVLLNVLSKCAGSKKFRPAPAAAFARECRGFYFELQELKEDDYYGI
TLSDDSDHQFLLANQVVVHQCGYIKEKNKSEFSWLDFVVTKFSDINDKIIPVFQENTLIG
VKLEDFEDWCKVAKLIEEKKHLTESGLDEIKKIKLNMNKGRVF*

>WT I-GpeMI
MGPTRNESINPWVLTGFADAEGSFILRIRNNNKSSAGYSTELGFQITLHKKDISILENIQS
TWKVGVIANSGDNAVSLKVTRFEDLRVVLNHFEKYPLITQKLGDYLLFKQAFSVMENKE
HLKIEGIKRLVGIKANLNWGLTDELKEAFVASGGENIFVASGGERSLINKNIPNSGWLAG
FTSGEGCFFVSLIKSKSKLGVQVQLVFSITQHARDRALMDNLVTYLGCGYIKEKKKSEF
SWLEFVVTKFSDIKDKIIPVFQVNNIIGVKLEDFEDWCKVAKLIEEKKHLTESGLEEIRNIK
LNMNKGRVL*

>I-GpeMI/VMA1 (GC1)
MGPTRNESINPWVLTGFADAEGSFILRIRNNNKSSAGYSTELGFQITLHKKDISILENIQS
TWKVGVIANSGDNAVSLKVTRFEDLRVVLNHFEKYPLITQKLGDYLLFKQAFSVMENKE
HLKIEGIKRLVGIKANLNWGLTDELKEAFVASGGENIFVASGGERSLINKNIPNSGWLAG
FTSGEGCFAKGTNVLMADGSIECIENIEVGNKVMGKDGRPREVIKLPRGRETMYSVVQ
KSQHRAHKSDSSREVPELLKFTCNATHELVVRTPRSVRRLSRTIKGVEYFEVITFEMGQ
KKAPDGRIVELVKEVSKSYPISEGPERANELVESYRKASNKAYFEWTIEARDLSLLGSH
VRKATYQTYAPILYENDHFFDYMQKSKFHLTIEGPKVLAYLLGLWIGDGLSDRATFSVDS

RDTSLMERVTEYAEKLNLCAEYKDRKEPQVAKTVNLYSKVVRGNGIRNNLNTENPLWD
AIVGLGFLKDGVKNIPSFLSTDNIGTRETFLAGLIDSDGYVTDEHGIKATIKTIHTSVRDGL
VSLARSLGLVVSVNAEPAKVDMNGTKHKISYAIYMSGGDVLLNVLSKCAGSKKFRPAPA
AAFARECRGFYFELQELKEDDYYGITLSDDSDHQFLLANQVVVHNCFFVSLIKSKSKLG
VQVQLVFSITQHARDRALMDNLVTYLGCGYIKEKKKSEFSWLEFVVTKFSDIKDKIIPVF
QVNNIIGVKLEDFEDWCKVAKLIEEKKHLTESGLEEIRNIKLNMNKGRVL*

>I-GpeMI/VMA1 (GC2)
MGPTRNESINPWVLTGFADAEGSFILRIRNNNKSSAGYSTELGFQITLHKKDISILENIQS
TWKVGVIANSGDNAVSLKVTRFEDLRVVLNHFEKYPLITQKLGDYLLFKQAFSVMENKE
HLKIEGIKRLVGIKANLNWGLTDELKEAFVASGGENIFVASGGERSLINKNIPNSGWLAG
FTSGEGCFFVSLIKSKSKLGVQVQLVFSITQHARDRALMDNLVTYLGCFAKGTNVLMAD
GSIECIENIEVGNKVMGKDGRPREVIKLPRGRETMYSVVQKSQHRAHKSDSSREVPEL
LKFTCNATHELVVRTPRSVRRLSRTIKGVEYFEVITFEMGQKKAPDGRIVELVKEVSKSY
PISEGPERANELVESYRKASNKAYFEWTIEARDLSLLGSHVRKATYQTYAPILYENDHFF
DYMQKSKFHLTIEGPKVLAYLLGLWIGDGLSDRATFSVDSRDTSLMERVTEYAEKLNLC
AEYKDRKEPQVAKTVNLYSKVVRGNGIRNNLNTENPLWDAIVGLGFLKDGVKNIPSFLS
TDNIGTRETFLAGLIDSDGYVTDEHGIKATIKTIHTSVRDGLVSLARSLGLVVSVNAEPAK
VDMNGTKHKISYAIYMSGGDVLLNVLSKCAGSKKFRPAPAAAFARECRGFYFELQELK
EDDYYGITLSDDSDHQFLLANQVVVHNCGYIKEKKKSEFSWLEFVVTKFSDIKDKIIPVF
QVNNIIGVKLEDFEDWCKVAKLIEEKKHLTESGLEEIRNIKLNMNKGRVL*

>WT I-PanMI
MGFKRNFSTLESKLNPSYISGFVDGEGSFMLTIIKDNKYKLGWRVVCRFVISLHKKDLSL
LNKIKEFFDVGNVFLMTKDSAQYRVESLKGLDLIINHFDKYPLITKKQADYKLFKMAHNLI
KNKSHLTKEGLLELVAIKAVINNGLNNDLSIAFPGINTILRPDTSLPQILNPFWLSGFVDAE
GCFSVVVFKSKTSKLGEAVKLSFILTQSNRDEYLIKSLIEYLGCGNTSLDPRGTIDFKVTN
FSSIKDIIVPFFIKYPLKGNKNLDFTDFCEVVRLMENKSHLTKEGLDQIKKIRNRMNTNRK
*

>I-PanMI/VMA1 (GC1)
MGFKRNFSTLESKLNPSYISGFVDGEGSFMLTIIKDNKYKLGWRVVCRFVISLHKKDLSL
LNKIKEFFDVGNVFLMTKDSAQYRVESLKGLDLIINHFDKYPLITKKQADYKLFKMAHNLI
KNKSHLTKEGLLELVAIKAVINNGLNNDLSIAFPGINTILRPDTSLPQILNPFWLSGFVDAE
GCFAKGTNVLMADGSIECIENIEVGNKVMGKDGRPREVIKLPRGRETMYSVVQKSQHR
AHKSDSSREVPELLKFTCNATHELVVRTPRSVRRLSRTIKGVEYFEVITFEMGQKKAPD
GRIVELVKEVSKSYPISEGPERANELVESYRKASNKAYFEWTIEARDLSLLGSHVRKATY
QTYAPILYENDHFFDYMQKSKFHLTIEGPKVLAYLLGLWIGDGLSDRATFSVDSRDTSLM
ERVTEYAEKLNLCAEYKDRKEPQVAKTVNLYSKVVRGNGIRNNLNTENPLWDAIVGLGF
LKDGVKNIPSFLSTDNIGTRETFLAGLIDSDGYVTDEHGIKATIKTIHTSVRDGLVSLARSL
GLVVSVNAEPAKVDMNGTKHKISYAIYMSGGDVLLNVLSKCAGSKKFRPAPAAAFAREC
RGFYFELQELKEDDYYGITLSDDSDHQFLLANQVVVHNCFSVVVFKSKTSKLGEAVKLS
FILTQSNRDEYLIKSLIEYLGCGNTSLDPRGTIDFKVTNFSSIKDIIVPFFIKYPLKGNKNLD
FTDFCEVVRLMENKSHLTKEGLDQIKKIRNRMNTNRK*

>I-PanMI/VMA1 (GC2)
MGFKRNFSTLESKLNPSYISGFVDGEGSFMLTIIKDNKYKLGWRVVCRFVISLHKKDLSL

LNKIKEFFDVGNVFLMTKDSAQYRVESLKGLDLIINHFDKYPLITKKQADYKLFKMAHNLI
KNKSHLTKEGLLELVAIKAVINNGLNNDLSIAFPGINTILRPDTSLPQILNPFWLSGFVDAE
GCFSVVVFKSKTSKLGEAVKLSFILTQSNRDEYLIKSLIEYLGCFAKGTNVLMADGSIECI
ENIEVGNKVMGKDGRPREVIKLPRGRETMYSVVQKSQHRAHKSDSSREVPELLKFTC
NATHELVVRTPRSVRRLSRTIKGVEYFEVITFEMGQKKAPDGRIVELVKEVSKSYPISEG
PERANELVESYRKASNKAYFEWTIEARDLSLLGSHVRKATYQTYAPILYENDHFFDYMQ
KSKFHLTIEGPKVLAYLLGLWIGDGLSDRATFSVDSRDTSLMERVTEYAEKLNLCAEYK
DRKEPQVAKTVNLYSKVVRGNGIRNNLNTENPLWDAIVGLGFLKDGVKNIPSFLSTDNI
GTRETFLAGLIDSDGYVTDEHGIKATIKTIHTSVRDGLVSLARSLGLVVSVNAEPAKVDM
NGTKHKISYAIYMSGGDVLLNVLSKCAGSKKFRPAPAAAFARECRGFYFELQELKEDDY
YGITLSDDSDHQFLLANQVVVHNCGNTSLDPRGTIDFKVTNFSSIKDIIVPFFIKYPLKGN
KNLDFTDFCEVVRLMENKSHLTKEGLDQIKKIRNRMNTNRK*

Figure S3. Amino acid sequences of homing endonucleases and TSMs.

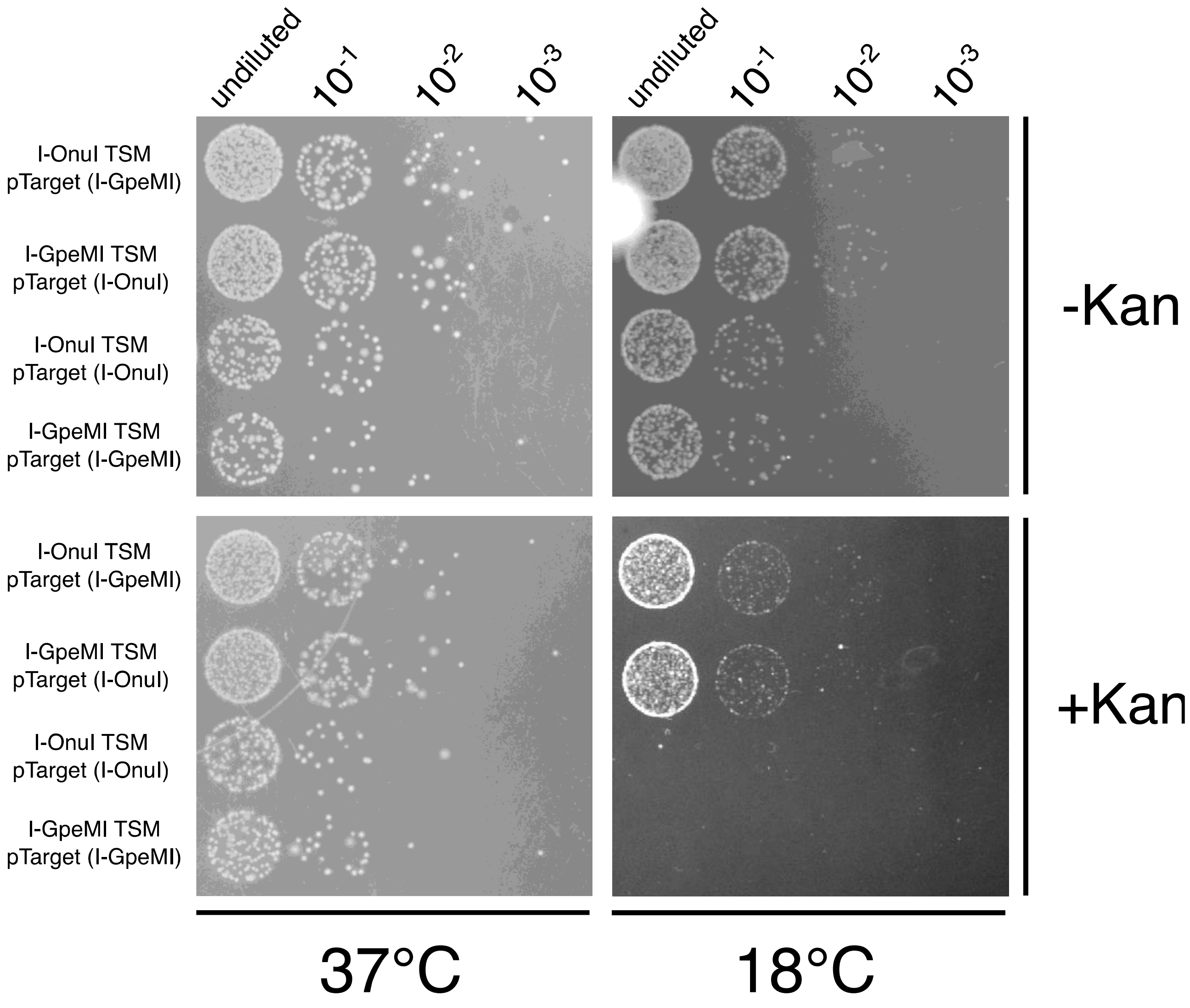

Figure S4. Cleavage preference orthogonality of I-OnuI and I-GpeMI. Cleavage is indicated by the failure to grow on kanamycin-supplemented media, resulting from the loss of pTarget.

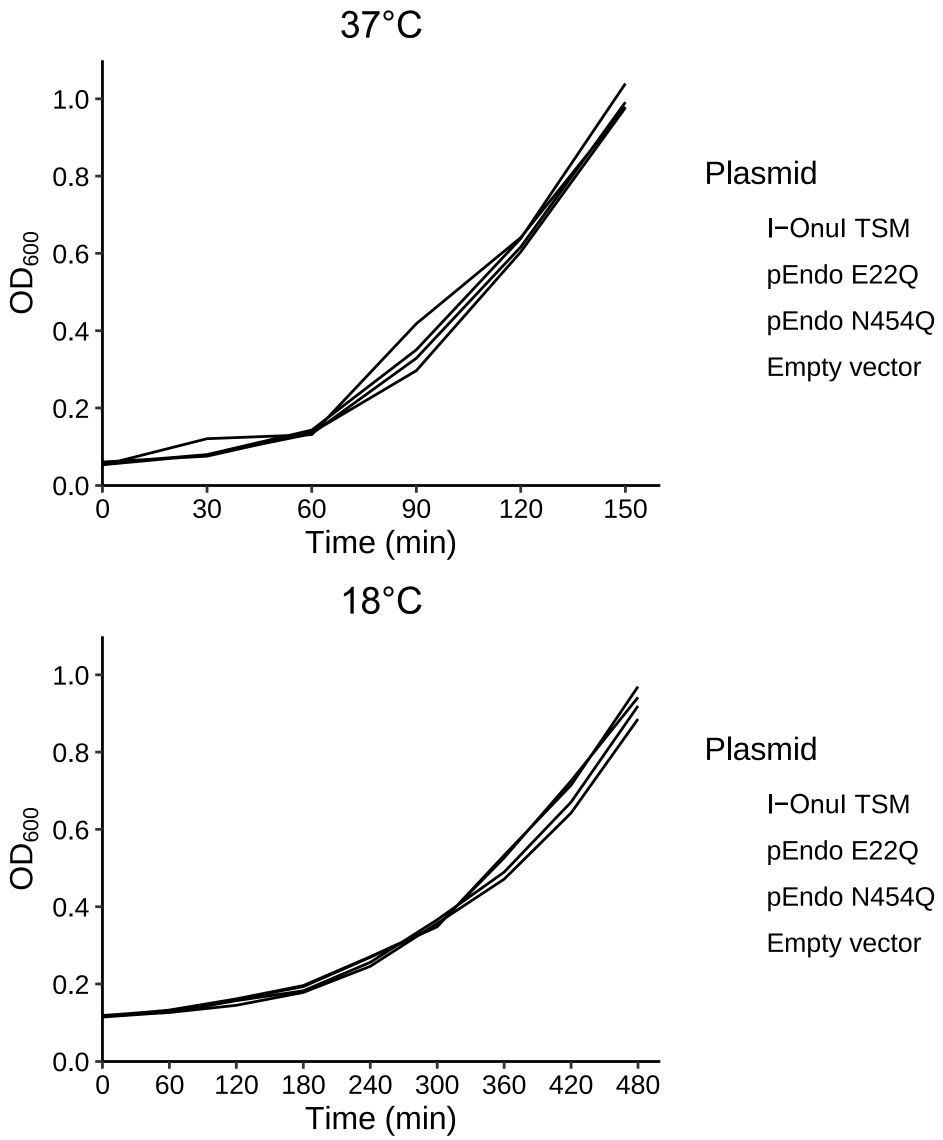

Fig S5. Growth curve assays of *E. coli* Nissle 1917 carrying I-OnuI TSM or various negative controls. Each point is an individual replicate (n=3).

A

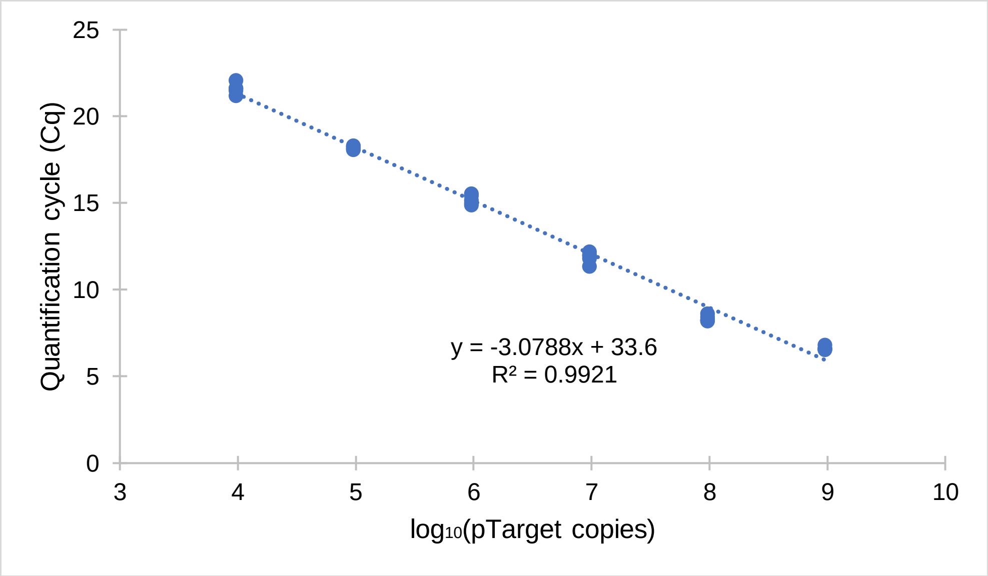

B

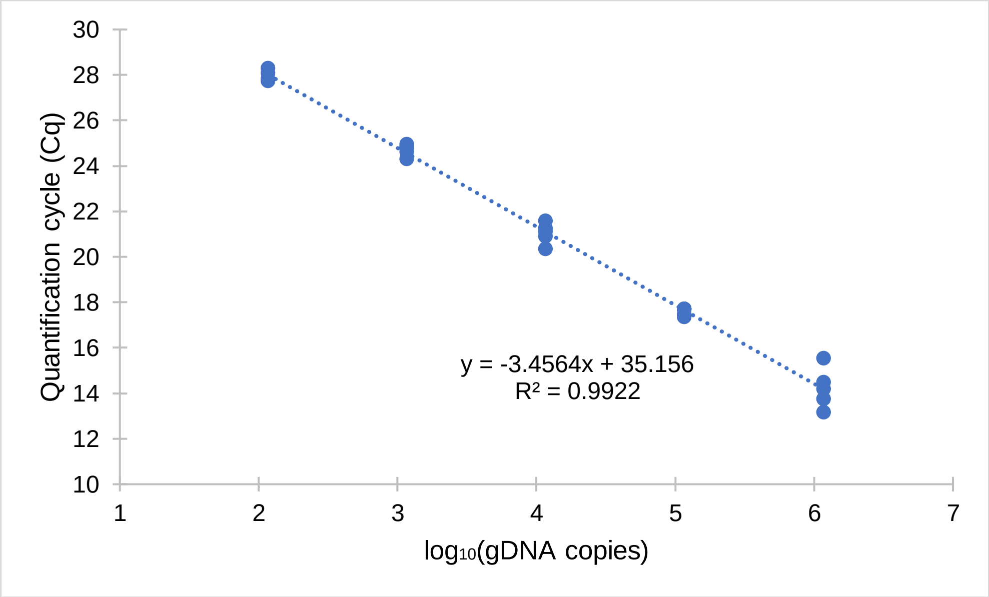

Fig S6. Standard curves of serial dilutions of pTarget (A) and *E. coli* Nissle 1917 gDNA (B) for qPCR. qPCR was performed with SYBR Select Master Mix (Applied Biosystems) on the Viia 7 (ThermoFisher Scientific). The primer pair (DE-7269 and DE-7270) targeting pTarget amplified a 150 bp amplicon on the kanamycin resistance gene (primer efficiency 111%). The primer pair (DE-7271 and DE-7272) targeting the *CspA* gene on the *E. coli* Nissle 1917 chromosome also amplified a 150 bp amplicon (primer efficiency 94.7%).

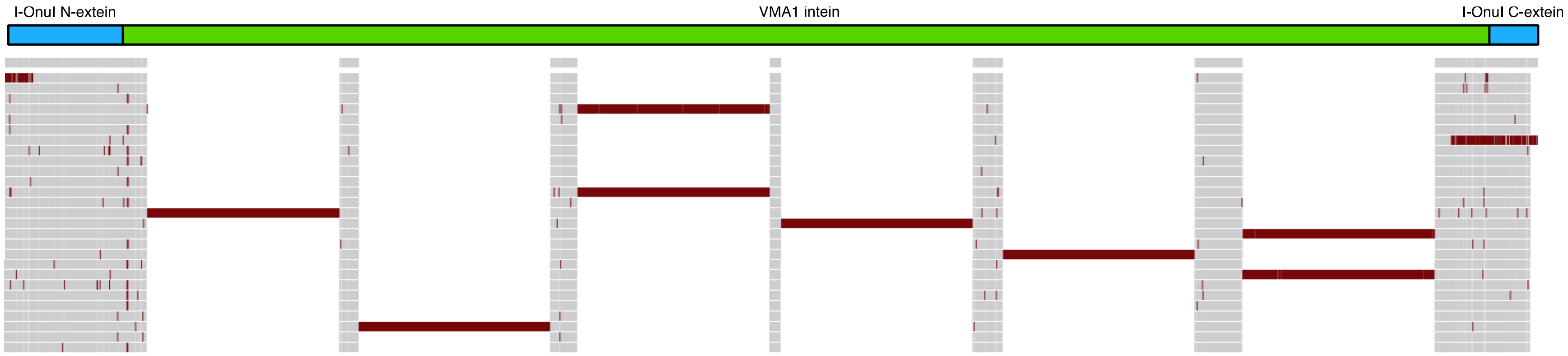

Fig. S7. Alignment of the I-OnuI CDS against the pEndo plasmid isolated from 27 escape mutants. Local pairwise (MAFFT) alignment, using the Smith-Waterman algorithm, performed and visualized in Benchling. Top bar is the reference sequence, every other bar is the CDS from one of the escape mutants. Red bands indicate mismatches, either SNPs or the IS911 insertion sequence at various sites in the I-OnuI TSM CDS.

```
     Wild-type: .....CAAGGGTACCAATGTTTTAATGGCGGATGGGTCTATTGAATGTATTGAAAACATTGAGGTTG.....

12-bp deletion: .....CAAGGGTACCAATGTTTTAATGGCG------------GAATGTATTGAAAACATTGAGGTTG.....
```

Figure S8. 12-bp VMA1 intein deletion product resulting in frequent I-OnuI TSM inactivation. Highlighted in red are the palindromic inverted repeat regions thought to contribute to the observed frequency of the deletion.

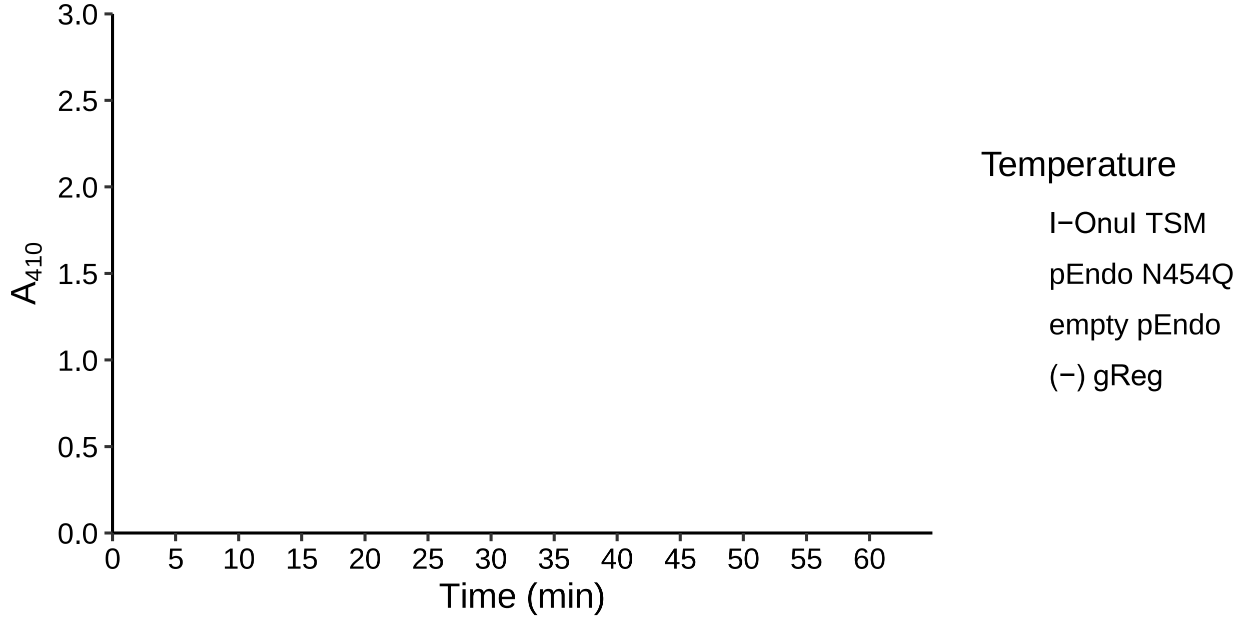

Fig S9. Production of the 4-nitrophenol cleavage product by GusA. *E. coli* Nissle 1917 carrying pTarget-gReg and pEndo with either active TSM or various negative controls were permeabilized. The formation of 4-nitrophenol was determined by measuring the absorbance at 410 nm every minute for 1 hour in a BioTek Epoch 2 Microplate Spectrophotometer. Each data point is an individual replicate (n=3).
